# Supplementary material for: Patient-reported experience measure (PREM) for patients with interstitial lung disease (ILD): modification of a pre-existing measure
Source: BMJ Open Respir Res. 2026 Jan 20;13(1):e003330. doi: 10.1136/bmjresp-2025-003330 (PMC12820880; doi:10.1136/bmjresp-2025-003330)
Supplement: online supplemental file 1 [file bmjresp-13-1-s001.docx]

**Supplementary material Table S4: Distribution of ILD PREM responses by number of respondents**

| **1. Your needs and preferences** | | | | | | | | |
| --- | --- | --- | --- | --- | --- | --- | --- | --- |
| Statements | Strongly Agree | Agree | Neither Agree nor Disagree | Disagree | Strongly Disagree | Not Applicable | No Answer Recorded | Total |
| *a) During my appointments, I felt that I was treated respectfully as an individual* | 64 | 9 |  |  |  |  |  | 73 |
| *b) I was involved as much as I wanted to be in decisions about my treatment and care* | 53 | 16 | 2 |  |  |  | 2 | 73 |
| *c) My personal circumstances and preferences were taken into account when planning and deciding on my treatment and care* | 53 | 14 | 4 |  |  | 1 | 1 | 73 |
| *d) I was given information in a way that I could understand* | 59 | 12 | 1 |  |  |  | 1 | 73 |
| *e) I was given enough information to help me make decisions about my treatment* | 56 | 13 | 3 |  |  |  | 1 | 73 |
| **2. Co-ordination of care and communication / care across departments** | | | | | | | | |
| *a) I was made aware that there is a team of health professionals looking after me* | 61 | 11 |  |  |  |  | 1 | 73 |
| *b) When I needed help, I was able to access different members of my health team* | 34 | 10 | 4 | 1 |  | 22 | 2 | 73 |
| *c) There is a member of my health team who can help me to see other specialists in the team if I need to* | 48 | 18 | 6 |  |  | 1 |  | 73 |
| *d) I feel that the people I see at the clinic are fully up to date with my current situation* | 54 | 14 | 3 |  |  |  | 2 | 73 |
| **3. Information, education and self-care** | | | | | | | | |
| *a) I feel that I was given information at the time I needed it* | 54 | 17 | 2 |  |  |  |  | 73 |
| *b) I feel that I have a good understanding of the treatments I am on or being offered* | 44 | 27 | 2 |  |  |  |  | 73 |
| *c) I have been told about patient organisations or groups that can help me* | 51 | 21 |  |  |  |  | 1 | 73 |
| *d) I have been offered an opportunity to attend a self-management programme suitable to my needs* | 39 | 16 | 10 | 2 |  | 6 |  | 73 |
| **4. Daily living and physical comfort** | | | | | | | | |
| *a) I feel that I have the right support to adapt and manage my daily activities around my changing lung condition* | 42 | 17 | 10 |  |  |  | 4 | 73 |
| *b) If my symptoms get much worse (e.g. I experience an exacerbation or flare), I have been able to get help quickly* | 24 | 9 | 8 | 1 |  | 23 | 8 | 73 |
| **5. Emotional Support** | | | | | | | | |
| *a) I feel able to approach a member of my health team to discuss any worries about my condition and my treatment or their effect on my life* | 43 | 20 | 4 |  | 1 | 1 | 4 | 73 |
| *a) I feel able to approach a member of my health team to discuss any worries about my condition and my treatment or their effect on my life* | 43 | 18 | 5 |  |  | 2 | 5 | 73 |
| **6. Family and Friends** | | | | | | | | |
| *a) I feel able to include members of my family in my appointments, if I want to, to become involved in decisions about my care* | 54 | 12 | 5 |  |  |  | 2 | 73 |
| **7. Access to care** | | | | | | | | |
| *a) At appointments, I feel that I have enough time with the healthcare professional to cover everything I want to discuss* | 53 | 17 | 1 |  |  |  | 2 | 73 |
| **Statements** | **Yes** | **No** | **Up to 1 week** | **1 to 3 weeks** | **3 to 6 weeks** | **6 to 12 weeks** | **over 12 weeks** | **No Answer** |
| *b) I have had appointments cancelled unexpectedly* | 13 | 53 |  |  |  |  |  | 7 |
| *c) If yes, how long have you had to wait for a new appointment?* |  |  | 3 | 4 | 4 | 1 |  | 1 |
| *d) I have needed extra treatment or a change of treatment (between routine clinic appointments)* | 6 | 57 |  |  |  | 1 |  | 9 |
| *e) If yes, how long did it take for this to happen?* |  |  | 2 | 3 | 1 |  |  | 3 |
| **8. Overall experience of care** | | | | | | | | |
| **Statements** | **Strongly Agree** | **Agree** | **Neither Agree nor Disagree** | **Disagree** | **Strongly Disagree** | **Not Applicable** | **No Answer Given** | **Total** |
| *a) Overall, in the past year, I have had a good experience of care for my lung condition* | 37 | 18 | 9 | 1 |  | 5 | 3 | 73 |
