# Supplementary material for: Patient-reported experience measure (PREM) for patients with interstitial lung disease (ILD): modification of a pre-existing measure
Source: BMJ Open Respir Res. 2026 Jan 20;13(1):e003330. doi: 10.1136/bmjresp-2025-003330 (PMC12820880; doi:10.1136/bmjresp-2025-003330)
Supplement: online supplemental file 2 [file bmjresp-13-1-s002.pdf]

Table S5: ILD-PREM

Section	Statement	Strongly agree	Agree	Neither agree nor disagree	Disagree	Strongly disagree	Not applicable
1.Your needs and preferences	a) During my appointments, I felt that I was treated respectfully as an individual	○	○	○	○	○	
	b) I was involved as much as I wanted to be in decisions about my treatment and care	○	○	○	○	○	
	c) My personal circumstances and preferences were taken into account when planning and deciding on my treatment and care	○	○	○	○	○	
	d) I was given information in a way that I could understand	○	○	○	○	○	
	e) I was given enough information to help me make decisions about my treatment	○	○	○	○	○	
2.Co-ordination of care and communication<br><br>Care across departments	a) I was made aware that there is a team of health professionals looking after me	○	○	○	○	○	○
	b) When I needed help, I was able to access different members of my health team	○	○	○	○	○	○
	c) There is a member of my health team who can help me to see other specialists in the team if I need to	○	○	○	○	○	○
	d) I feel that the people I see at the clinic are fully up to date with my current situation	○	○	○	○	○	
3. Information, education and self-care	a) I feel that I was given information at the time I needed it	○	○	○	○	○	
	b) I feel that I have a good understanding of the treatments I am on or being offered	○	○	○	○	○	
	c) I have been told about patient organisations or groups that can help me	○	○	○	○	○	
	d) I have been offered an opportunity to attend a self-management programme suitable to my needs	○	○	○	○	○	○
4. Daily living and physical comfort	a) I feel that I have the right support to adapt and manage my daily activities around my changing lung condition	○	○	○	○	○	

	**b) If my symptoms get much worse (e.g. I experience an exacerbation or flare), I have been able to get help quickly**	○	○	○	○	○	○
**5. Emotional support**	**a) I feel able to approach a member of my health team to discuss any worries about my condition and my treatment or their effect on my life**	○	○	○	○	○	
	**b) I feel able to talk about personal or intimate issues with my health team, if I want to**	○	○	○	○	○	
**6. Family and friends**	**a) I feel able to include members of my family in my appointments, if I want to, to become involved in decisions about my care**	○	○	○	○	○	
**7. Access to care**	**a) At appointments, I feel that I have enough time with the healthcare professional to cover everything I want to discuss**	○	○	○	○	○	
	**b) I have had appointments cancelled unexpectedly**	○ Yes	○ No				
	c) If yes, how long have you had to wait for a new appointment?	○ Up to 1 week	○ 1 to 3 weeks	○ 3 to 6 weeks	○ 6 to 12 weeks	○ Over 12 weeks	
	**d) I have needed extra treatment or a change of treatment (between routine clinic appointments)**	○ Yes	○ No				
	e) If yes, how long did it take for this to happen?	○ Up to 1 week	○ 1 to 3 weeks	○ 3 to 6 weeks	○ 6 to 12 weeks	○ Over 12 weeks	
**8. Overall experience of care**	**a) Overall in the past year, I have had a good experience of care for my lung condition**	○	○	○	○	○	
